# Multi-objective optimization of coronary stent using Kriging surrogate model

**DOI:** 10.1186/s12938-016-0268-9

**Published:** 2016-12-28

**Authors:** Hongxia Li, Junfeng Gu, Minjie Wang, Danyang Zhao, Zheng Li, Aike Qiao, Bao Zhu

**Affiliations:** 1 School of Mechanical Engineering, Dalian University of Technology, Dalian, 116023 Liaoning China; 2 State Key Laboratory of Structural Analysis for Industrial Equipment, Department of Engineering Mechanics, Dalian University of Technology, Dalian, 116023 Liaoning China; 3 College of Life Science and Bioengineering, Beijing University of Technology, Beijing, 100124 China; 4 School of Materials Science and Engineering, Dalian University of Technology, Dalian, 116023 Liaoning China

**Keywords:** Stent, Dogboning, Radial elastic recoil, Black-box techniques, Kriging surrogate model, Design optimization

## Abstract

**Background:**

In stent design optimization, the functional relationship between design parameters and design goals is nonlinear, complex, and implicit and the multi-objective design of stents involves a number of potentially conflicting performance criteria. Therefore it is hard and time-consuming to find the optimal design of stent either by experiment or clinic test. Fortunately, computational methods have been developed to the point whereby optimization and simulation tools can be used to systematically design devices in a realistic time-scale. The aim of the present study is to propose an adaptive optimization method of stent design to improve its expansion performance.

**Methods:**

Multi-objective optimization method based on Kriging surrogate model was proposed to decrease the dogboning effect and the radial elastic recoil of stents to improve stent expansion properties and thus reduce the risk of vascular in-stent restenosis injury. Integrating design of experiment methods and Kriging surrogate model were employed to construct the relationship between measures of stent dilation performance and geometric design parameters. Expected improvement, an infilling sampling criterion, was employed to balance local and global search with the aim of finding the global optimal design. A typical diamond-shaped coronary stent-balloon system was taken as an example to test the effectiveness of the optimization method. Finite element method was used to analyze the stent expansion of each design.

**Results:**

27 iterations were needed to obtain the optimal solution. The absolute values of the dogboning ratio at 32 and 42 ms were reduced by 94.21 and 89.43%, respectively. The dogboning effect was almost eliminated after optimization. The average of elastic recoil was reduced by 15.17%.

**Conclusion:**

This article presents FEM based multi-objective optimization method combining with the Kriging surrogate model to decrease both the dogboning effect and radial elastic recoil of stents. The numerical results prove that the proposed optimization method effectively decreased both the dogboning effect and radial elastic recoil of stent. Further investigations containing more design goals and more effective multidisciplinary design optimization method are warranted.

## Background

As the leading cause of mortality, cardiovascular disease is often related to atherosclerosis which caused by the progressive formation of plaque and eventually results in an obstruction (stenosis) for blood flow through the artery [1–5]. Compared to traditional treatments such as drugs and surgery for coronary artery diseases (narrowing or blockage of the coronary arteries), percutaneous transluminal coronary stenting with the aid of coronary balloon angioplasty is more widely adopted in clinical practice thanks to its high initial success rate, minimal invasive nature, and improved long-term effectiveness. A stent is a wire metal meshed tube placed in the vessel during coronary balloon angioplasty to offer radial strength and to overcome the acute elastic recoil. The stent is put over a balloon catheter and moved into stenosis segment. Then, it expands as the balloon is inflated to open the blocked vessel. After the balloon and catheter are removed, the stent remains in the vessel to act as a scaffold to help prevent arteries from becoming narrowed or blocked again. Nowadays, intravascular stents are routinely and successfully used in medical treatment, but it still needs to be improved. For example, in-stent restenosis remains the main obstacle for the development of stent. It is known that in-stent restenosis is caused by artery injury due to stent expansion and vascular inflammation to the stent struts. Therefore, scholarly efforts to improve stent expansion performance and reduce the injury of blood vessel caused by stent implantations in stent design optimization are of great importance.

A desirable stent should possess a number of excellent mechanical properties, including smaller dogboning ratio and smaller radial elastic recoil. The dogboning phenomenon caused by non-uniform balloon-stent expansion has a significant impact on the development of thrombus and intimal hyperplasia [6, 7]. A larger dogboning ratio indicates a more serious warpage at the ending struts, which will cause mechanical damage to the vessel wall and results in in-stent restenosis [8–10]. Additionally, the radial elastic recoil due to elastic deformation of stent has a significant impact on the mechanical support of stent. It is believed that the stent design may affect stent expansion performance such as the dogboning ratio and radial elastic recoil. Thus, it is important to predict the dogboning effect and radial elastic recoil and to optimize the design before manufacturing the stent.

Computational simulation (e.g., finite element analysis (FEA)) can be a very useful tool to study the stent expansion [11–14]. Dumoulin and Cochelin [11] evaluated and characterized the mechanical properties and behaviors of a balloon expandable stent. Etave et al. [15] compared the mechanical performance of two types of stents. In terms of stent design, Migliavacca et al. [16, 17] and Beule et al. [1] assessed the mechanical properties and behavior of balloon expandable stents to determine how the FEA method could be used to optimize stent designs. It is easy to study the mechanical properties and analyze the effective factors, but it is difficult to find the globally optimal solution since the functional relationship between the geometrical parameters and dilation performance of stent is complex, nonlinear and implicit. For the traditional stent design method, a finite number of different designs are assessed and compared with each other to find an optimal design. This method is often used in industry when commercial demands restrict the time spent on developing a better product. However a limited number of discrete points fail to represent all the information in the design space and thus it is very difficult to find the optimal design through the traditional methods. For traditional methods, the designer has to formulate how many different designs can be tested in the available time and their work is also affected by the design methodology, problem fidelity, parameterization and measures of performance. The focus of the study is on the computational design of stent. However, since the stent is minute and boundary conditions for the expanding of stent in the vessel are complicated, it is relatively hard to apply finite element modelling in the optimization of stent. Actually not only the dependences mentioned above but also the availability and power of computers and software shall be considered when the traditional design methods are adopted.

Consequently, some approximation models are widely used in engineering to construct simplified approximations for analysis codes, especially when the analysis is hard and time-consuming. Incorporating a shape optimization algorithm based on a proven convergence theory into the design process allows engineers to systematically identify the most favourable designs. An adaptive optimization method based on Kriging surrogate model was already proposed to eliminate the dogboning phenomenon by Li et al. [18]. A derivative-free optimization algorithm coupling computational fluid dynamics (CFD) was used for stent design by Gundert et al. [19]. In this paper, Kriging models were used as alternatives to the method of traditional second-order polynomial response surfaces for constructing global approximations in stent optimization. As a semi-parametric approach, the Kriging model [20, 21] is much more flexible than approaches based on parametric behavioral models.

Taking the consideration above in mind, we adopted the Kriging model to create an approximate functional relationship between the design objective and design parameters to replace the expensive reanalysis of the stent dogboning ratio and radial elastic recoil. The optimization iterations are based on the approximate relationship between the design objective and design parameters to reduce the high computational cost. An adaptive optimization method based on the Kriging surrogate model combing with modified rectangular grid (MRG) approach was proposed to minimize the radial elastic recoil and the dogboning effect of stent during the expansion process. Expected improvement (EI) function is employed in the adaptive process [18], which can balance local and global searches and then find the global optimal design even with a small sample size. The FEA solver of ANSYS was used to analyze the measurements of stent expansion performance.

## Methods

### Finite element model

**Fig. 1:**
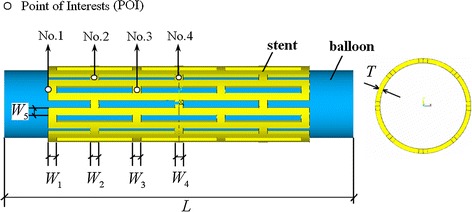
Balloon-stent system model and design variables: *W*_*i,*_ *i* = 1, …, 5, *T* and *L*, where *W*_*i*_ is the width of struts, *T* is the thickness of stent, *L* is the length of balloon. Four POIs used to validate the FEA simulation for radial recoil calculation

**Fig. 2:**
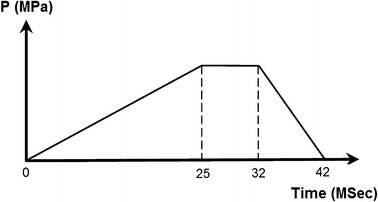
Time-related pressure which include three load phases: 0–25 ms linear loading; 25–32 ms constant loading; 32–42 ms linear unloading

A typical Palmaz-Schatz stent (shown in Fig. 1) was investigated in this study. The geometries and loading method of the stent was supported by Ref. [22]. A balloon that is 11.4 mm in length and 0.12 mm in thickness was placed inside the stent. The outer wall of the balloon is close to the inner wall of the stent. Geometric dimensions of the stent are shown in Fig. 1. A time-related pressure (shown in Fig. 2) was loaded on the inner surface of balloon to stimulate the expanding process of the balloon- stent system.

Bi-linear elastic–plastic and hyper-elastic (Mooney-Rivlin) materials were assumed for slotted tube stents and balloon. Data of the material properties used in this study was from previous studies [23, 22].

**Fig. 3:**
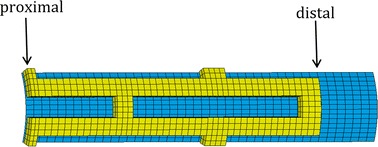
Finite element mesh. The balloon consisted of 660 elements with 44 elements along its length and 15 element in circumference and the stent consisted of 672 elements with 48 elements along its modelled length and 14 elements in circumference

**Fig. 4:**
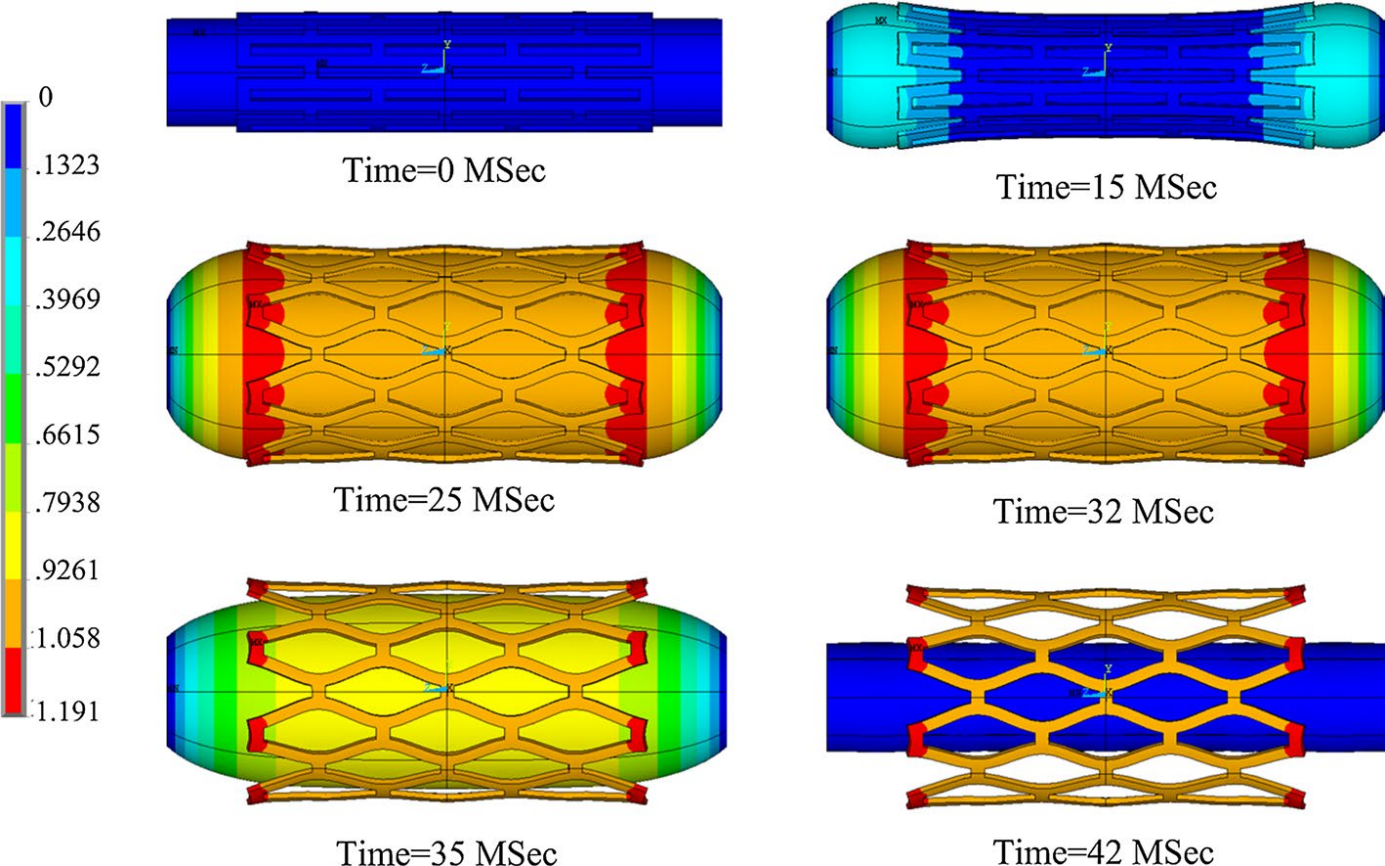
Stent expansion process symmetrical displayed in the circumferential direction

The balloon-stent dilatation involves nonlinearities namely contact, elasto-plasticity and large deformation. Thence, “solid 185” in ANSYS was used for three-dimensional modeling of the stent. The element possesses plasticity, large deflection, large strain capabilities and mixed formulation capability for stimulating deformation of nearly incompressible elasto-plastic materials. Shell 181 was used to model the balloon since it is well-suited to be applied in cases that involve large strain nonlinear. Due to the symmetry of the entire structure and the loading pressure only, 1/16 of the model (1/8 in circumferential direction and 1/2 in longitudinal direction) was preformed to analyze the dogboning ratio and radial recoil of stent, as shown in Fig. 3. The balloon was modelled as a hyperelastic shell. The nodes at the distal end of balloon were constrained without rigid body displacement, while the nodes at the distal end of stent were free. Symmetry boundary conditions were applied to the symmetry parts of the stent and balloon. Pressure involving three load phases (shown in Fig. 2) was applied to the inner surface of the balloon. A face-to-face contact between balloon and stent inner surface was considered and the friction between them was ignored. The stent expanded as the balloon was inflated, as shown in Fig. 4. The stent was expanded to a large permanent deformation due to the expansion of balloon and the plastic strain occurs in most parts of struts. Thence, the stent can stay in the stenotic artery permanently and holds the artery wall open to prevent restenosis or narrowing of coronary arteries after the balloon is deflated and withdrawn. In this study, the stent outer diameter of each design was dilated to a same diameter of 4.54 mm. Obviously, the pressures were varied with different stent geometries. The binary-search method was adopted to find the pressure of each design of stent to dilate the proximal ends of it (marked in Fig. 3) to the nominal diameter after unloading of the balloon. This is to allow the stenotic segment to be opened in agreement with a health artery (diameter 4.54 mm in this study) after stent dilation.

### Optimization problem

Generally, the dogboning effect exists throughout the expanding process and usually reaches its maximum in the beginning of loading [24], but the struts have no direct contact with the vessel wall. While, during the stent deformed from 25 to 32 ms, the dogboning ratio was relatively large [18] in this study and it could cause injury to the vascular wall. Therefore, the dogboning ratio of the stent can be defined as:

1 $${\text{Dogboning}}\,{\text{ratio (}}DR )= \frac{{d_{\text{radial}}^{\text{distal}} - d_{\text{radial}}^{\text{proximal}} }}{{d_{\text{radial}}^{\text{proximal}} }}$$

where $d_{\text{radial}}^{\text{distal}}$ and $d_{\text{radial}}^{\text{proximal}}$ denote the distal and proximal radial displacements respectively when it is at 32 ms. It is easy to find that when the distal of stent is over-expanded, *DR* is larger than 0; while when the proximal of stent is over-expanded, *DR* is less than 0. Therefore, in order to expand the stent uniformity along the longitudinal direction, we need to minimize the absolute value of *DR*.

For the typical diamond stent, bridge struts provide the main radial support of stenosis. Furthermore, the radial displacement and radial recoil of stent in these bridge struts are different only in the case that there is a uniform expansion of stent along its longitudinal direction. Therefore, four points on the bridge struts were chosen as the points of interest (shown in Fig. 1), and the average of radial recoil at these four points was taken as stent radial elastic recoil. Therefore, the stent radial elastic recoil can be defined as:

2 $${\text{Radial elastic recoil (}}RER )= \frac{1}{4}\sum\limits_{i = 1}^{4} {\frac{{R_{{{\text{POI(No}}.i )}}^{\text{loading}} - R_{{{\text{POI(No}}.i )}}^{\text{unloading}} }}{{R_{{{\text{POI(No}} .i )}}^{\text{loading}} }}}$$

where $R_{{{\text{POI(No}}.i )}}^{\text{loading}}$ and $R_{{{\text{POI(No}}.i )}}^{\text{unloading}}$ are the radius of the stent at the point of interest (POI) (shown in Fig. 1) when it is at 32 and 42 ms respectively. Obviously, *RER* is the average of the radial elastic recoil at the POIs.

Our optimization objective is to find a set of design variables to reduce both *DR* and *RER*. A common approach in multi-objective optimization is to compute the weighted sum of all the objective functions. Therefore, the multi-objective problem can be transformed into a single-objective problem and the objective function can be considered as:

3 $$\omega_{1} \left| {DR} \right| + \omega_{2} RER$$

Because the two objectives are mutually incompatible, it is not easy to choose appropriate weights *ω*_1_ and *ω*_2_. Moreover, the two objectives have different scales. If we scale both |*DR*| and *RER* to the same range namely [0, 1], then we might be able to assign some reasonable weights. In order to do this, we need to improve the objective function. And the optimization of the coronary stent can be defined as follows:

4 $$\begin{aligned} & Min{\text{ }}f({\mathbf{x}}) = \omega \frac{{\left| {DR} \right| - \left| {DR} \right|_{{\min }} }}{{\left| {DR} \right|_{{\max }} - \left| {DR} \right|_{{\min }} }} + (1 - \omega )\frac{{RER - RER_{{\min }} }}{{RER_{{\max }} - RER_{{\min }} }} \hfill \\ & S.t.{\text{ }}\,\,0.22 \le W_{i} \le 0.34, \quad i = 1,\ldots, 4 \\ {\text{ }}& \qquad0.2 \le W_{5} \le 0.3 \hfill \\ {\text{ }}& \qquad0.1 \le T \le 0.14 \hfill \\ {\text{ }}& \qquad4.5 \le L \le 6.5 \hfill \\ \end{aligned}$$

where *ω* ∈ (0, 1), |*DR*|_min_ and |*DR*|_max_ are the minimum and maximum of |*DR*| in the samples respectively, *RER*_min_ and *RER*_max_ are the minimum and maximum of *RER* in the samples respectively. **x** is the design variables consisting of the geometrical parameters *W*_*i*_, *i*= 1,…, 5 and T of stent and L (the length of balloon), as shown in Fig. 1. In this study, *ω* = 0.5.

### Optimization method

A finite element based multi-objective optimization method combining with Kriging surrogate model [18] was constructed for the stent optimization to improve stent expansion performance. Kriging was used to build the approximate functional relationship between the design objective and design variables. A modified rectangular grid (MRG) approach was adopted to generate the initial sample for Kriging. EI function was employed to balance the local and global search to find the global optimal design.

#### Kriging approximate method

The Kriging model is regarded as a method of functional modeling of a stochastic process. Therefore it is named as the “stochastic process model”, which is written as:

5 $$\mathop y\limits^{ \wedge } ({\mathbf{x}}^{i} ) = \text{F} (\beta ,{\mathbf{x}}^{i} ) + \text{z} ({\mathbf{x}}^{i} ) = \text{f}^{T} ({\mathbf{x}}^{i} ){\varvec{\upbeta}} + \text{z} ({\mathbf{x}}^{i} )$$

where, **x** ^*i*^ = {*x*_1_^*i*^, *x*_2_^*i*^,…, *x*_*m*_^*i*^} denotes the *i*th sample point with a number of *m* variables; $\hat{y}({\mathbf{x}}^{i})$ is an approximate function fitted to a number of *n* sample points; f(**x** ^*i*^) is a linear or nonlinear function of **x** ^*i*^; **β** is the regression coefficient to be estimated; and z(**x** ^*i*^) is the stochastic function, with a mean of zero and a variance *σ* ^2^. The spatial correlation function between stochastic functions is given by

6 $$\text{corr} \left[ {\text{z} ({\mathbf{x}}^{i} ),\text{z} ({\mathbf{x}}^{j} )} \right] = \text{R} (\theta ,{\mathbf{x}}^{i} ,{\mathbf{x}}^{j} ) = \coprod\limits_{l = 1}^{m} {\exp \left[ { - \theta (x_{l}^{i} - x_{l}^{j} )^{2} } \right]}$$

where R(*θ*, **x** ^*i*^, **x** ^*j*^) is the Gaussian correlation function with *θ*, which characterizes the spatial correlation between two samples. Parameters can be estimated by maximizing the likelihood of sample points.

7 $$\begin{array} {l} \hat{\sigma }^{2} = \frac{{({\mathbf{y}} - {\mathbf{f}}^{{\rm T}} {\varvec{\hat{\upbeta }}})^{{\rm T}} \text{R} ^{{ - 1}} ({\mathbf{y}} - {\mathbf{f}}^{{\rm T}} {\varvec{\hat{\upbeta }}})}}{n} \hfill \\ {\varvec{\hat{\upbeta }}} = \frac{{{\mathbf{f}}^{{\rm T}} \text{R} ^{{ - 1}} {\mathbf{y}}}}{{{\mathbf{f}}^{{\rm T}} \text{R} ^{{ - 1}} {\mathbf{f}}}} \hfill \\ \hat{\theta } = \min \left\{ {\psi \left( {\mathbf{\theta }} \right) \equiv \left| \text{R} \right|^{{\frac{1}{{n_{s} }}}} \sigma ^{2} } \right\} \hfill \\ \end{array}$$

where *f* = [*f*_1_, *f*_2_, …, *f*_*n*_]. The estimates $\hat{\beta }$ and $\hat{\sigma }^{2}$ can be obtained by Eq. (7).

#### Predictor

A linear combination of the response values of sample **Y** could be used to estimate $\hat{y}({\mathbf{x}}^{*} )$ of a new point x*

8 $$\hat{y}({\mathbf{x}}^{ * } ) = {\mathbf{c}}^{\rm T} {\mathbf{Y}}$$

The mean squared error (MSE) of this predictor can be minimized by unbiased estimation, which gives

9 $$\hat{y}\left( {{\mathbf{x}}^{ * } } \right) = {\mathbf{f}}\left( {{\mathbf{x}}^{ * } } \right){\varvec{\hat{\upbeta }}} + {\mathbf{r}}\left( {{\mathbf{x}}^{ * } } \right)^{\rm T} {\varvec{\upgamma}}$$

where

10 $$\begin{aligned} &{\mathbf{\gamma = R}}^{{ - {\mathbf{1}}}} \left( {{\mathbf{Y}} - {\varvec{{\text{F}}\hat{\upbeta }}}} \right) \hfill \\ {\mathbf{r}}\left( {{\mathbf{x}}^{\text{*}} } \right) &\,\,= \left[ {{\text{R}}\left( {{\varvec{\uptheta}}\text{,}\,\,\,{\mathbf{x}}_{{\mathbf{1}}} \text{,}\,{\mathbf{x}}^{{\mathbf{*}}} } \right),\, \ldots {\text{R}}\left( {{\varvec{\uptheta}}\text{,}\,\,{\mathbf{x}}_{{\mathbf{n}}} \text{,}\,{\mathbf{x}}^{ *} } \right)} \right] \hfill \\ \end{aligned}$$

Therefore, the function value $\hat{y}\left( {{\mathbf{x}}^{ * } } \right)$ at every new point x* can be predicted by using Eq. (9).

#### Sampling strategy

MRG approach was adopted to generate the sample points for constructing the Kriging model. The range of *m* design variables was defined as $l_{j} \le x_{j} \le u_{j} ,\,\,j = 1,\, \ldots ,\,m$. The number of levels in the jth dimension is *q*_*j*_. Then the approach is performed as follows:

1. Narrow the range of variables as
  11 $$l_{j} \le x_{j} \le \hat{u}_{j} ,\,\,\hat{u}_{j} = u_{j} - \frac{1}{2}\frac{{u_{j} - l_{j} }}{{q_{j} - 1}},\quad j = 1,\, \ldots ,\,m$$
2. Perform rectangular grid (RG) sampling [25] in the narrowed space as
  12 $$x_{j}^{i} = l_{j} + k_{j}^{(i)} \frac{{\hat{u}_{j} - l_{j} }}{{q_{j} - 1}},\quad k_{j} = 0,\,\,1,\, \ldots ,\,q_{j} - 1 \quad i = 1,2,\, \ldots, \, \prod\limits_{j = 1}^{m} {q_{j} }$$
3. Add a stochastic movement of each sample point in each dimension as
  13 $$\frac{{\alpha_{ij} }}{2}\frac{{u_{j} - l_{j} }}{{q_{j} - 1}} \quad j = 1,\,2,\, \ldots ,\,m$$

where ${\kern 1pt} {\kern 1pt} {\kern 1pt} \alpha_{ij} \in [ 0, 1]$, which is assumed to be normally distributed.

Compared to RG, MRG has several advantages such as preventing sample points from lying in boundary, which can provide more useful information for constructing the Kriging model. What’s more, MRG can ensure that the points have lower pairwise correlation between the factors and avoid the case of sample points spaced too close to each other. The distance between two arbitrary points must satisfy

14 $$d{\kern 1pt} {\kern 1pt} {\kern 1pt} {\kern 1pt} \ge {\kern 1pt} {\kern 1pt} {\kern 1pt} {\kern 1pt} \mathop {min}\limits_{1 \le j \le m} {\kern 1pt} {\kern 1pt} {\kern 1pt} {\kern 1pt} \left[ {\frac{{u_{j} - l_{j} }}{{2(q_{j} - 1)}}\left( {1 - \frac{1}{{q_{j} - 1}}} \right)} \right]$$

#### Expected improvement (EI)

Generally, the response surface based optimization is to find the minimum of the response surface. But this method often results in a local minimum, even if iterations are performed in the search. Fortunately, an “expected improvement (*EI*)” function can be used to balance local and global search, which regarded as an effective global optimization (EGO) [21]. The *EI* method computes the extent of improvement of response value. For any point ***x*** which is not one of the already known sample points in the design space, the value of *Y*(***x***) is unknown. Thus, *Y*(***x***) can be considered as a random variable and assumed it is normally distributed with a mean $\hat {\mathbf{y}} ({\mathbf{x}})$ and variance σ^2^ got from the Kriging predictor. If the current best optimization function value is *Y*_min_, and *Y*(*x*) = *Y*_min_ − *I*, then an improved *I* will be obtained. The likelihood to achieve such an improvement is given by the normal density function

15 $$\frac{1}{{\sqrt {2\pi \sigma ({\mathbf{x}})} }}\exp \left[ {\frac{{\left( {Y_{{\min }} - I - \hat{y}({\mathbf{x}})} \right)}}{{2\sigma ^{2} ({\mathbf{x}})}}} \right]$$

The expected improvement is the expected value of the improvement obtained by integrating over the following density:

16 $$\text{E} [I({\mathbf{x}})] = \int_{I = 0}^{I = \infty } {I\left\{ {\frac{1}{{\sqrt {2\pi } \sigma ({\mathbf{x}})}}\exp \left[ { - \frac{{(\text{Y}_{ {\rm min} } - I - \hat{y}({\mathbf{x}}))^{2} }}{{2\sigma^{2} ({\mathbf{x}})}}} \right]} \right\}dI}$$

With integration by parts, it can be obtained

17 $$\text{E} [I({\mathbf{x}})] = \sigma ({\mathbf{x}})[u\Phi (u) + \phi (u)]$$

where $\Phi$ and $\phi$ denote the normal cumulative distribution and density functions respectively. And

18 $$u = \frac{{Y_{_\text{{min}} } - \hat{y}({\mathbf{x}})}}{{\sigma ({\mathbf{x}})}}$$

where Eq. (18) can be meaningless if σ(x) equals zero. Hence, it can be written as

19 $$\text{E} (I) = \left\{ \begin{array} {ll} \sigma ({\mathbf{x}})[u\Phi (u) + \phi (u)] &\quad {\text{if }}\sigma ({\mathbf{x}}) > 0 \hfill \\ 0 &\quad\,{\text{if }}\sigma ({\mathbf{x}}) = 0 \hfill \\ \end{array} \right.$$

The first term of Eq. (19) refers to the difference between the current minimum response value *Y*_min_ and the prediction $\hat{y}\left( {\mathbf{x}} \right)$ at **x**. Hence, it is large when $\hat{y}\left( {\mathbf{x}} \right)$ is small. The second term is the product of the root mean squared error (RMSE) σ(**x**) and the normal density function $\phi (u)$. $\phi (u)$ is large when σ(**x**) is large and $\hat{y}\left( {\mathbf{x}} \right)$ is closed to *Y*_min_. Thus, the expected improvement will be larger when the predicted value is smaller than *Y*_min_ and/or there is a lot of uncertainty associated with the prediction.

#### The convergence criterion

The optimization iteration stops when

20 $$\begin{aligned} \frac{{EI_{k} ({\mathbf{x}})}}{{Y_{_\text{{max}} } - Y_{_\text{{min}} } }} \le \Delta_{1} \hfill \\ \left| {f_{k} ({\mathbf{x}}) - f_{k - 1} ({\mathbf{x}})} \right| \le \Delta_{2} \hfill \\ \left| {f_{k} ({\mathbf{x}}) - \hat{y}_{k} ({\mathbf{x}})} \right| \le \Delta_{3} \hfill \\ \end{aligned}$$

where Δ_1_, Δ_2_ and Δ_3_ are the convergence tolerances. *Y*_max_ and *Y*_min_ are the maximal and minimal function values of sample points respectively. *f*_*k*_ and *f*_*k*−1_ are the objective function values at the *k*th and *k* − 1th iteration, respectively. This stopping criterion enjoys the advantage that the user can set the “relative” tolerance Δ_1_ without considering the magnitude of the problem response.

### Implementation of optimization procedure

The optimization algorithm for a coronary stent based on Kriging model combining DOE methods and *EI* function can be described as follows:

Step 1: Get a set of *n*_*s*_ samples using MRG.
Step 2: Run ANSYS program with Binary-search method to dilate stent at sample point *i, i* = 1,…, *n*_*s*_ to nominal diameter and obtain $d_{\text{radial}}^{\text{distal}}$ and $d_{\text{radial}}^{\text{proximal}}$, $R_{{{\text{POI(No}}.i )}}^{\text{loading}}$ and $R_{{{\text{POI(No}}.i )}}^{\text{unloading}}$, *i* = 1, …, 4. Calculate *f*(**x**_*i*_) in problem (3) at the sample point *i, i* = 1, …, *n*_*s.*_
Step 3: Find the sample point with the minimum *f*(**x**_*i*_) as the initial point for the optimization.
Step 4: Get an approximate functional relationship between the design objective *f*(**x**) and design variables using Kriging surrogate model based on the trial samples. Calculate *EI*(**x**) based on *f*(**x**).
Step 5: Select optimization algorithm to implement the optimization design based on max *EI* and obtain the modified design **x**_*k.*_
Setp 6: Get the predictive value $\hat{y}_{k}$ of **x**_*k*_ based on Kriging and compute *f*(**x**_*k*_) by ANSYS program.
Step 7: The optimization iteration was stopped when a suitable level of convergence is reached and/or the available time for the optimization process is exhausted. The process of constructing and maximizing *EI* does not stop until the Euclidean norm between real value *f*(**x**_*k*_) and predictive value $\hat{y}_{k}$ fall below a given tolerance, the Euclidean norm between current and previous iterates falls below a given tolerance, and the criterion stipulated in “The convergence criterion” section is reached. If not, then add the modified design into the set of samples and go to step 3.

## Results

**Fig. 5:**
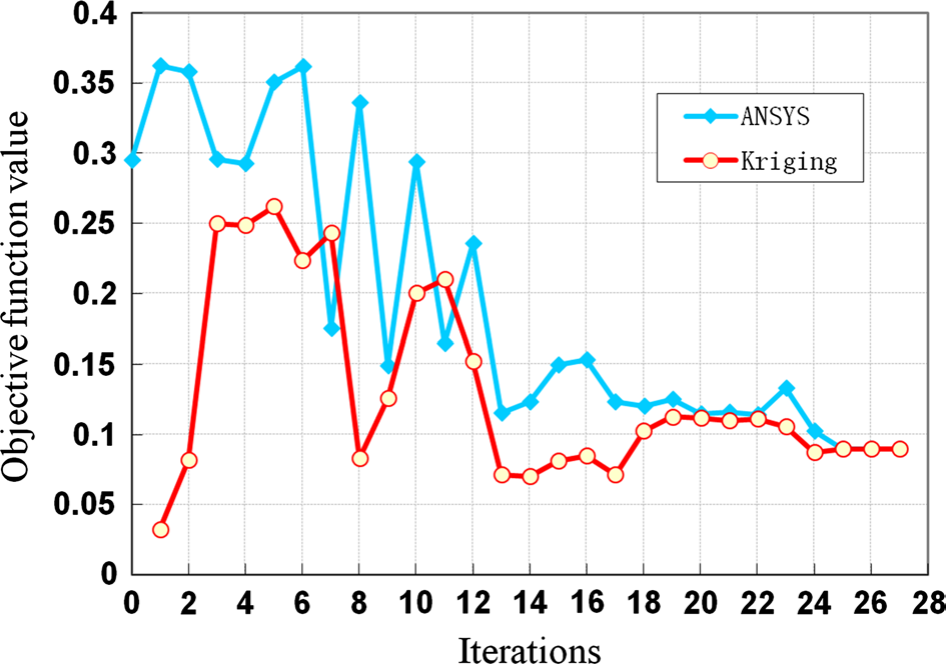
Optimization iteration process. 0–25 ms: expanded gradually; 25–32 ms: fully expanded; 35–42 ms: recoil

The absolute value of dogboning ratio and radial elastic recoil of Palmaz-Schatz stent were minimized by the multi-objective optimization method proposed in this paper. The initial trial samples which included the initial design and 30 samples generated by MRG (listed in Table 1) were selected to construct the Kriging surrogate model. The dogboning ratio and radial elastic recoil of stents for all trial samples are simulated by finite element method. *EI* function was adopted to balance local and global search in the design space. The optimization process started from the initial point which has the minimum value of design objective among all the sample points. 27 iterations were needed to obtain the optimal solution as shown in Fig. 5.

**Table 1:** Initial training sample points selected by MRG

Samples	*W*_1_	*W*_2_	*W*_3_	*W*_4_	*W*_5_	T	L	P	*DR*		*RER*
	(mm)	(mm)	(mm)	(mm)	(mm)	(mm)	(mm)	(MPa)	32 ms	42 ms	
1	0.309	0.2587	0.2703	0.2355	0.2935	0.1245	4.6935	2.0988	0.2495	0.2518	0.0177
2	0.3168	0.3013	0.3323	0.2819	0.2065	0.1323	5.6613	1.8081	0.0002	0.0002	0.0205
3	0.2781	0.3129	0.2316	0.2239	0.2258	0.1052	5.2742	1.7864	0.1537	0.1568	0.0205
4	0.3129	0.2858	0.2277	0.2781	0.2806	0.1284	5.9839	1.7864	0.2010	0.2037	0.0162
5	0.3245	0.3323	0.2897	0.2935	0.2226	0.1258	5.8548	1.8289	0.0624	0.0634	0.0186
6	0.3052	0.2548	0.309	0.3245	0.2871	0.1168	6.3065	1.9537	0.3058	0.3107	0.0152
7	0.2239	0.3206	0.2355	0.251	0.271	0.1206	6.2419	1.8800	0.1500	0.1529	0.0158
8	0.2703	0.2819	0.3129	0.3168	0.2839	0.1026	5.0161	1.8908	0.1663	0.1681	0.0172
9	0.2587	0.3168	0.3361	0.2394	0.2516	0.1219	5.7258	1.9655	0.0434	0.0431	0.0186
10	0.2626	0.3361	0.3206	0.2974	0.2742	0.1116	4.8871	1.9850	0.2191	0.2234	0.0191
11	0.3284	0.2394	0.2548	0.3013	0.2581	0.1335	4.629	1.0199	0.2831	0.2874	0.0211
12	0.251	0.3052	0.3013	0.2742	0.2903	0.1361	5.9194	1.8888	0.0917	0.0918	0.0185
13	0.2742	0.2974	0.2394	0.2316	0.2484	0.1348	5.3387	1.9205	0.0656	0.0672	0.0194
14	0.2548	0.2665	0.2239	0.309	0.2194	0.1194	5.1452	1.8079	0.1861	0.1895	0.0217
15	0.2897	0.2703	0.2665	0.2897	0.2968	0.1039	6.371	1.9168	0.2538	0.2578	0.0151
16	0.3361	0.3284	0.2587	0.2703	0.2452	0.1077	5.5323	1.8502	0.0013	0.0022	0.0188
17	0.2432	0.2781	0.3284	0.2548	0.2129	0.1232	5.5968	1.7848	0.0498	0.0501	0.0194
18	0.2858	0.309	0.2781	0.3206	0.2097	0.1065	6.1129	1.7848	0.0580	0.0578	0.0198
19	0.2355	0.2626	0.2742	0.2277	0.2161	0.1155	6.0484	1.7490	0.0114	0.0125	0.0172
20	0.2471	0.2277	0.2819	0.2665	0.2677	0.1103	5.4677	1.8854	0.0402	0.0398	0.0177
21	0.2935	0.2316	0.2858	0.2587	0.2032	0.1271	4.8226	1.8120	0.2824	0.2865	0.0233
22	0.2394	0.2742	0.251	0.3052	0.2774	0.1374	4.9516	2.0552	0.1545	0.1556	0.0135
23	0.2316	0.251	0.3168	0.3129	0.2355	0.1297	6.1774	1.8380	0.1293	0.1307	0.0187
24	0.2974	0.3245	0.2626	0.3361	0.2419	0.131	4.5645	1.9760	0.3568	0.3630	0.0219
25	0.2277	0.2935	0.2471	0.3323	0.2323	0.1142	5.2097	1.8269	0.1689	0.1720	0.0204
26	0.3323	0.2897	0.3245	0.2626	0.2613	0.1129	4.7581	1.9655	0.2288	0.2322	0.0193
27	0.2819	0.2471	0.3052	0.2432	0.2548	0.1013	6.4355	1.7955	0.1695	0.1732	0.0166
28	0.2665	0.2239	0.2974	0.3284	0.2387	0.1181	5.7903	1.8350	0.0500	0.0515	0.0182
29	0.3013	0.2432	0.2935	0.2471	0.2645	0.1387	5.4032	2.0010	0.0348	0.0352	0.0182
30	0.3206	0.2355	0.2432	0.2858	0.229	0.109	5.0806	1.8160	0.1674	0.1697	0.0202

### Optimization results in details

The optimization result was compared to the original design as shown in Table 2. The absolute value of the dogboning ratio at 32 ms was reduced by 94.21%, which indicates that the dogboning effect was almost eliminated. Moreover, although the absolute value of dogboning ratio after unloading at 42 ms was not considered in the optimization objective, it was reduced by 89.43%. The average of radial elastic recoil was reduced by 15.17%. Because the radial elastic recoil is not only related to the stent’s structure, but also connected with the materials and expansion process of the stent, it is hard to eliminate the radial elastic recoil completely. Both the dogboning and radial elastic recoil are two important features to evaluate the stent expansion performance, but some factors that influence them are contradictory to each other. From the optimal result, we can see that the optimal stent with the greater *W*_5_ results in higher radial force, which means that the optimal stent can better support the artery wall. This explains the decrease of radial elastic recoil of optimal stent. But the stent with a higher radial force is hard to be expanded. Generally, the stent with a higher radial force is hard to be dilated and the ends of it will open first during expansion. This phenomenon is referred to as the dogboning effect. However, the smaller *W*_2_, *W*_3_, *W*_4_ and *T* together with appropriate *W*_1_ and *L* result in lower dogboning, although they lead to higher radial elastic recoil. It shows our proposed multi-objective optimization method can effectively find a set of design variables that minimizes both dogboning and elastic recoil.

**Table 2:** Optimization results

Stents	*W*_*1*_ (mm)	*W*_*2*_ (mm)	*W*_*3*_ (mm)	*W*_*4*_ (mm)	*W*_*5*_ (mm)	*T* (mm)	*L* (mm)	*P* (MPa)	*DR* (t = 32 ms)	*DR* (t = 42 ms)	*RER*
Original stent	0.28	0.28	0.28	0.28	0.249	0.12	5.8	1.8654	0.0622	0.0634	0.0178
Optimal stent	0.235	0.22	0.22	0.22	0.3	0.1	5.63	1.9077	0.0036	0.0067	0.0151

The absolute value of the dogboning ratio at 32 ms, when stent was fully expanded, was reduced by 94.21%. The absolute value of dogboning ratio at 42 ms, after stent recoil, was reduced by 89.43%. The average of radial elastic recoil was reduced by 15.17%

**Fig. 6:**
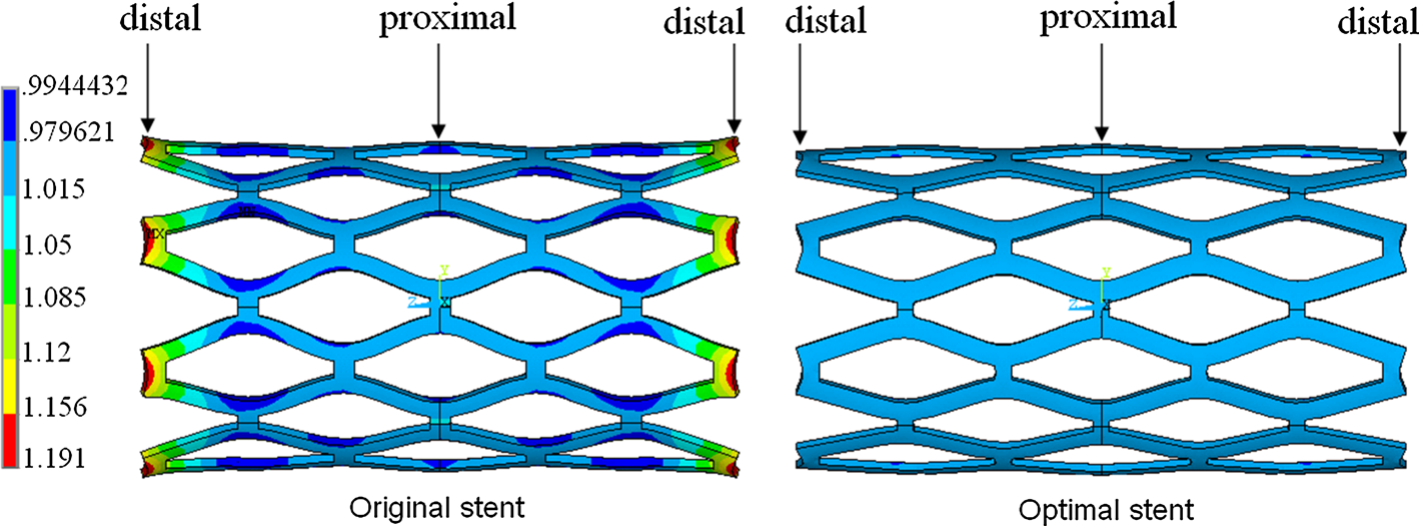
The radial displacement distributions of the original and optimal stents symmetrical displayed in the circumferential direction

The radial displacement distributions of the original and optimal stents at 32 ms, which symmetrical displayed in the circumferential direction, are shown in Fig. 6. The diameters of the original and optimal stents at the proximal ends were dilated to a same diameter of 4.54 mm after the deflation of balloon. The radial displacement of the original stent at the distal ends was much larger than that at the proximal ends. While, the proximal and distal radial expansions of the optimal stent were similar and the dogboning ratio was almost 0, which indicates uniform expansion along the length of optimal stent.

### Improvement of stent expansion process

**Fig. 7:**
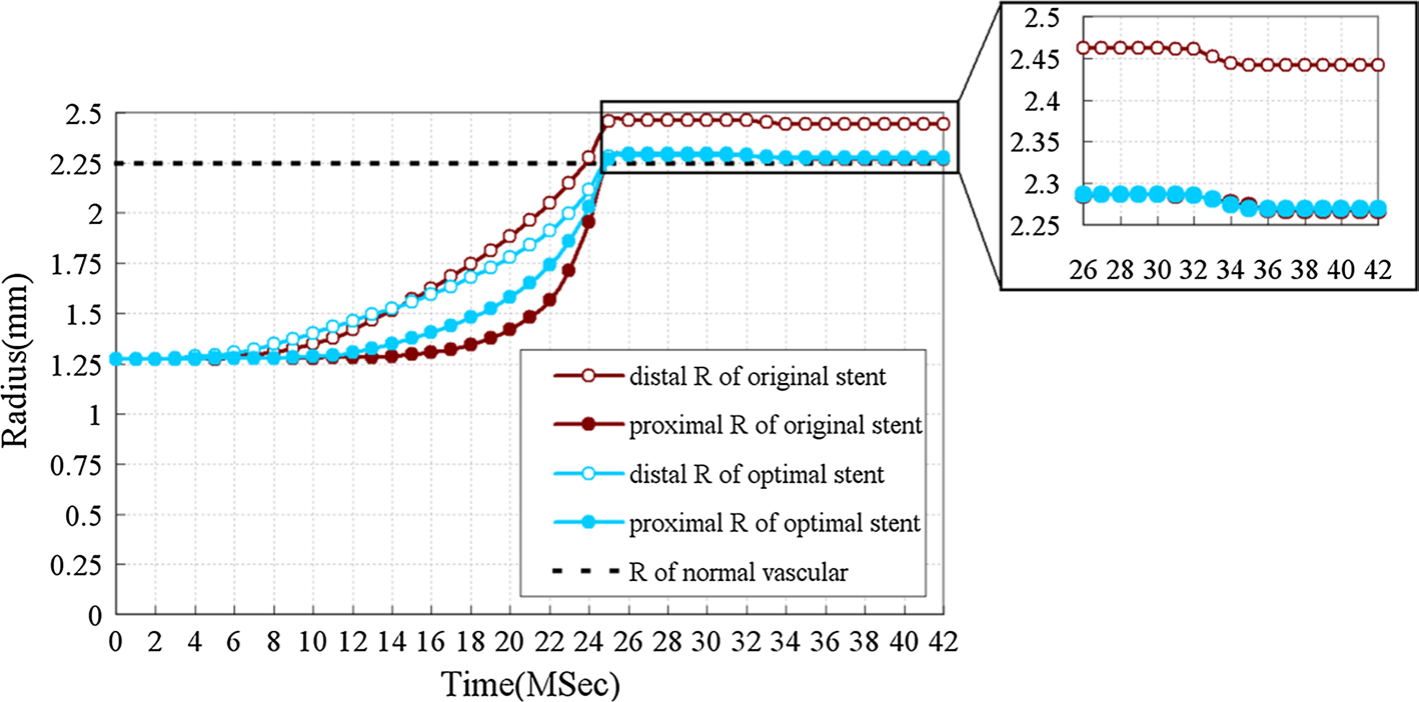
The proximal and distal radius of original and optimal stent in the dilation process. The radial of normal vascular is 2.25 mm. In first load phases 0–25 ms, both original and optimal stents were expanded gradually, but the struts didn’t reach the vessel wall until stents were fully expanded. In the second load phases 25–32 ms, the radius of the stents remained at a constant level. In the third load phases 32–42 ms, there was a small radial elastic recoil of stent, which occurred about 32–34 ms

The proximal and distal radius of both the original and optimal stents during the dilation process are shown in Fig. 7. The proximal radius of all the stents located at the sample points were expanded to the same nominal radius (2.27 mm) after the deflation of balloon. Figure 7 shows that the difference of the radius of optimal stent at the proximal and distal ends was smaller than that of the original one, particularly in the period from 25 to 32 ms. This indicates a uniform dilation of the optimal stent along its length. The reduction of the stent’s radius during the period from 25 to 42 ms demonstrates the radial elastic recoil of stent. Because the elastic recoil is not only related to the stent structure, but also related to the materials and expansion process, it wasn’t decreased completely in this study.

**Fig. 8:**
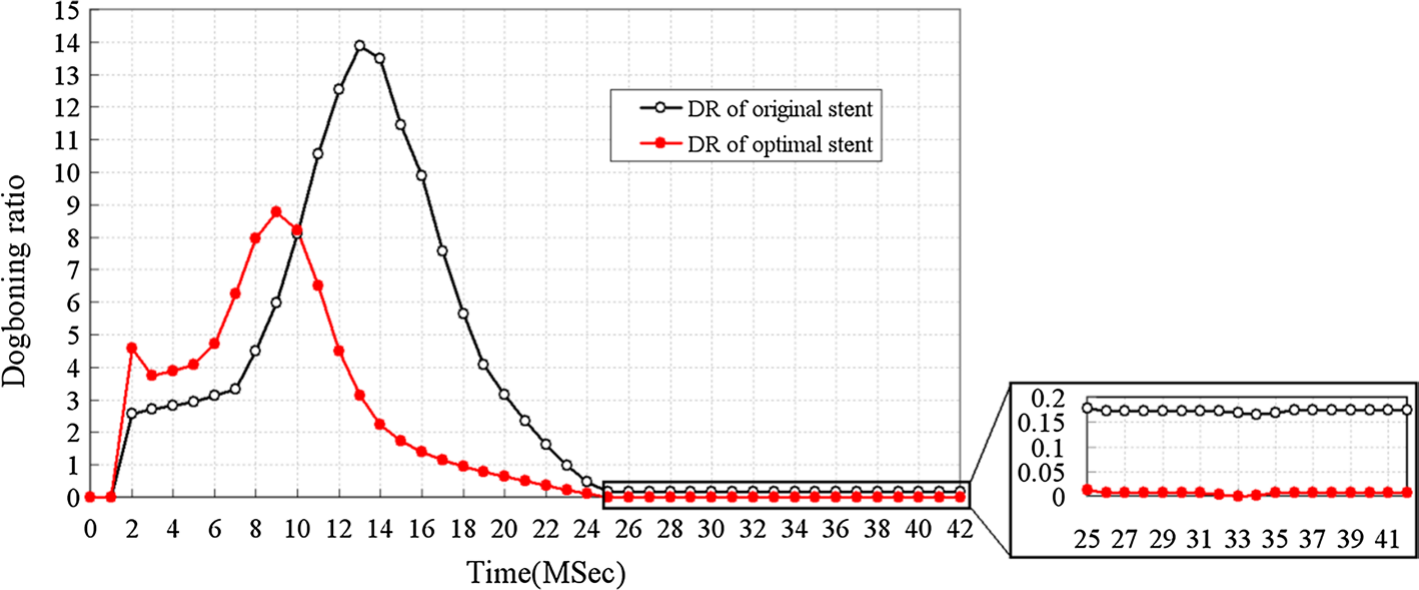
Dogboning ratio for original and optimal stents along with time (MSec) of stent dilation. The maximum *DR* of original stent is 13.9 at time = 13 ms, while the maximum *DR* of optimal stent is 8.78 at time = 9 ms. After time = 25 ms, *DR* of optimal stent is almost 0, while *DR* of original stent is about 0.17

Figure 8 shows the dogboning ratio for original and optimal stents along with time of stent dilation. The dogboning effect reached its maximum at the prophase of loading stage, and was reduced and remained in an almost constant value after stent expansion from 25 to 32 ms of loading and from 32 to 42 ms of unloading. The radial of stent during these periods reached its maximum, thereby the contact between stent and artery wall expanded and intensified. The dogboning effect during these periods would cause serious instantaneous mechanical damage to the blood vessel. The maximum *DR* of the optimal stent was 8.78, while the maximum *DR* of the original stent was 13.9. It means that the maximum *DR* has been reduced by 36.83% after optimization. Moreover, the time of maximum *DR* of optimal stent is earlier than that of original stent, which is helpful to reduce the risk of mechanical damage caused by stent on vessel wall. During the period from 25 to 42 ms, *DR* of the optimal stent was almost 0, while *DR* of the original stent was about 0.17. *DR* of the optimal stent in this period is decreased, and it denotes smaller instantaneous mechanical damage caused by stent on vessel wall.

## Discussions

A FEM based multi-objective optimization method combining with the Kriging surrogate model is proposed to reduce the dogboning effect and the radial elastic recoil of stent to improve the stent expansion performance. Our results show that the proposed optimization method could be used for stent design optimization effectively and conveniently. This provides a new method of stent design and represents a new direction of research. This optimization method combined with experimental verification can serve as a useful tool for stent design before manufacture.

In contrast to the expansive computational simulations employed in the comparison test studies [11, 12, 13, 15, 16, 17], the surrogate modeling approach in which response surface models (RSMs) were used to represent the relationship between design objectives and design variables [27]. Whilst most studies of stent design relate to multiple objectives, some articles only dealt with a single objective function. Harewood et al. [26] focused on radial stiffness of a single ring. Li et al. [28, 29] optimized stent dogboning and drug release, respectively. Grogan et al. [27] performed a single objective optimization for maximum radial strength. When considering multiple objectives, Pant et al. [30] and Bressloff [31] conducted FEA simulation to generate a range of multidisciplinary objectives. Pant et al. [30] constructed the Pareto fronts generated by treating each objective separately. Bressloff [31] recast the optimization as a constrained problem, wherein design improvement is sought in one objective while other objectives were considered as constraints. Multi-objective optimization of stent design involves a large number of design goals. It is difficult to find the optimal solution to improve all of them just by one of the methods to solve multi-objective problem, such as combining the design objectives in a single weighted objectives function, searching the Pareto fronts, and taking same design objectives as constraints. In future work, these methods can be used in combination under the premise of rational planning of design objectives and design variables of stent optimization systems, including stent auxiliary expansion, in-stent blood flow, drug release, and biomechanical response of vascular tissue, to improve the performance of stenting.

Some limitations of this study include: (a) FEA model of stent dilation does not contain blood vessels and thrombosis, (b) Balloon folding is not considered during its expansion process, (c) The results of optimal design have made some improvement of stenting performance, but it’s yet short of enough validation through experiment.

## Conclusions

This article presents a FEM based multi-objective optimization method combining with the Kriging surrogate model to decrease both the dogboning effect and radial elastic recoil of stents. The Kriging surrogate model coupled with DOE methods was adopted to construct an approximate functional relationship between the objective function and geometries. The *EI* function was employed to balance local and global searches with the aim of finding out the global optimal design. The proposed optimization method effectively decreased both the dogboning effect and radial elastic recoil of stent. More issues of stent design should be considered and more effective multidisciplinary design optimization method should be investigated to continue our study.
